# Supplementary material for: The macronuclear genomic landscape within Tetrahymena thermophila
Source: Microb Genom. 2024 Jan 11;10(1):001175. doi: 10.1099/mgen.0.001175 (PMC10868616; doi:10.1099/mgen.0.001175)
Supplement: Supplementary material 1 [file mgen-10-1175-s001.pdf]

Supplementary Material 1: DNA extraction electrophoresis

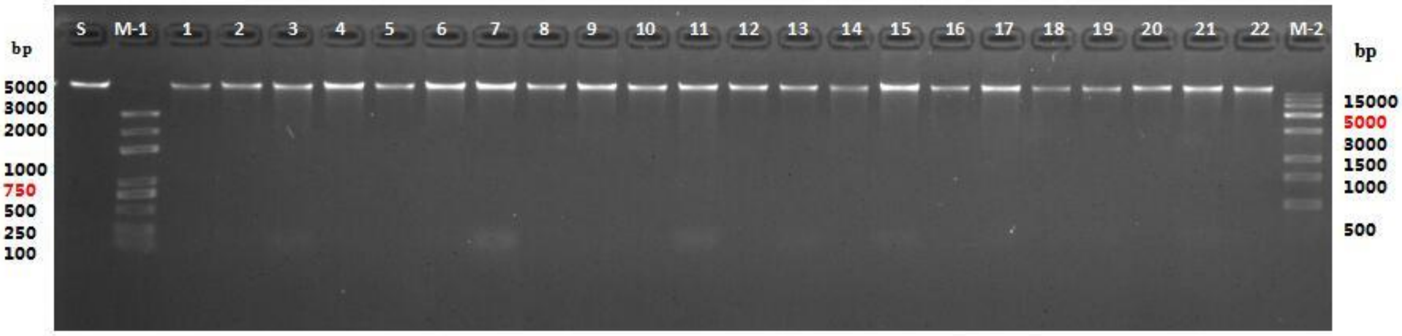

Electrophoresis of the 22 DNA extractions before sequencing. S is a positive control of DNA extraction, M-1 and M-2 are two different ladders, and strains D1 to D22 are indicated by the 1 to 22 numbers.

Supplementary Material 2: bioinformatics procedures

Modifications of the reference MAC SB210 genome assembly

The genome assembly of *T. thermophila* strain SB210 published in (Sheng, et al. 2020) was downloaded from http://ciliate.org. The assembly was first polished using Pilon v1.23 (Walker, et al. 2014) and the Illumina reads published in the same study (SRA database: SRR11906179) to correct potential sequencing/assembly errors. The two rounds of polishing generated 4027 and 55 modifications of the assembly (mostly one nucleotide indels).

We then renamed the 181 MAC chromosomes according to their respective origin in the 5 MIC chromosomes. The MAC chromosomes were mapped onto the MIC genome assembly [ref] downloaded from http://ciliate.org using minimap2 v2.17 (Li 2018) with the parameter '-x asm5'. For each MAC chromosome, the position in the MIC genome was defined as the centre of their mapping hits. The MAC chromosomes were finally named from 'chr_001' to 'chr_181' given these positions (chr_001 corresponding to the MAC chromosome located at the 5' end of the MIC chromosome 1). Finally, MIC chromosomes being centromeric, the MAC chromosomes corresponding to the MIC centromeres were identified by summing up the length of all MAC chromosomes of a given MIC chromosome as in (Hamilton, et al. 2016).

This corrected MAC genome assembly is referred in the text to as 'the SB210 genome assembly'.

Creation of a new predicted gene set

We noticed during our preliminary analyses that many of the genes defined in (Sheng, et al. 2020) were suspicious (e.g. presence of stop codons after genome polishing, unexpected large number of long introns). Therefore, we decided to create a new gene set, based mostly on the high quality Ensembl gene prediction (v2.2.48; 24,725 protein-coding genes and 927 ncRNA genes).

Firstly, the sequences of Ensembl protein-coding genes that did not have any ambiguous nucleotide (i.e. no 'N' in their sequence; 24,152 genes) were Blasted on to

the SB210 genome assembly with a 98% identity threshold (Blastn; v2.2.28). For each gene, the Ensembl prediction was transferred to the SB210 genome assembly using a custom python script if (i) the Blast hit covered the entire gene, (ii) there was no indel between the Blast hit and the gene sequence, (iii) the inferred exons did not overlap with previously defined exon positions and (iv) the predicted protein sequence inferred from the inferred exons did not contain any internal stop codon. These criteria led to the transfer of 23,643 protein-coding gene annotations to the SB210 genome assembly.

We then analysed the annotations of the protein-coding genes defined in (Sheng, et al. 2020) using two distinct approaches. Firstly, we transferred to the SB210 genome assembly the annotation of the genes with a name starting with 'TTHERM_' (e.g. genes initially defined by Ensembl) if (i) the inferred exons did not overlap with previously defined exon positions and (ii) the predicted protein sequence inferred from the inferred exons did not contain any internal stop codon. This step led to the transfer of 2,270 protein-coding gene annotations. Secondly, we transferred to the SB210 genome assembly the annotation of the genes with a name starting with 'g' (e.g. genes newly defined in (Sheng, et al. 2020); 919 genes) if they satisfied the two previous criteria and if their protein sequence had a blast hit (Blastp; v2.2.28) with a e-value below 1e-5 against the predicted proteome of either *Paramecium tetraurelia*, *Oxytricha trifallax* str. JRB310 or *Ichthyophthirius multifiliis* (alldownloaded from Ensembl Protist; https://protists.ensembl.org). This step led to the transfer of 105 protein-coding gene annotations, and a final gene set of 26,018 protein-coding genes.

Finally, the sequences of Ensembl ncRNA genes were Blasted on to the SB210 genome assembly with a 98% identity threshold (Blastn; v2.2.28). For each gene, the Ensembl prediction was transferred to the SB210 genome assembly if the Blast hit covered the entire ncRNA gene and did not overlap with any coding exon, leading to the annotation of 546 ncRNA genes.

Identification of MDS junctions

The corrected SB210 MAC genome assembly was aligned to the SB210 MIC genome assembly (Hamilton, et al. 2016), downloaded from http://ciliate.org, using minimap2 v2.17 (Li 2018) with the parameter '-x asm5'. Then, an in-house python script identified MDS junctions from the minimap2 output as follows:

_ only alignments with a score equal to 60 (i.e. maximal values) were considered for further analyses.

_ in-line MDS junctions were identified within alignments, corresponding to insertions in the MIC genome of at least 200 non-N nucleotides.

_ scrambled MDS junctions were identified between alignments, when (i) the positions of the extremity of two MAC alignments were separated by less than 10 bp (the position of the MDS junction corresponding to the average of the two extremity positions) and (ii) the corresponding MIC segment between the two extremities contained at least 200 non-N nucleotides.

A total of 9,973 MDS junctions were identified by this approach, including 7,273 ‘in-line’ MDS junctions (*i.e.*, the two MDS were adjacent and on the same strand in the MIC genome) and 2,700 ‘scrambled’ MDS junctions (*i.e.*, the two MDS were joined following a genomic rearrangement). This number of scrambled MDS junctions was very similar to that observed in (Sheng, et al. 2020) (2,711 scrambled MDS junctions). Over the 7,544 MDS junctions identified by (Hamilton, et al. 2016), 6,911 were found within 10-bp of one of the MDS junctions identified in this study. We thus consider our dataset reliable to assess intraspecific polymorphism patterns at MDS junctions.

Command lines for read mapping and SNP/indel calling

The program versions are indicated in Material and methods. The following command lines were repeated for each strain (D1 to D22). Firstly, trimmed reads were aligned to the reference genomes (MAC and mitochondrial genomes) and the alignments were filtered to retain high quality matches (i.e. with a mapping quality of at least 30):

- bwa mem -t 4 reference_genome.fa D1_trimmed.fq.gz D1_2_trimmed.fq.gz > D1.sam
- samtools view -q 30 -h -b -S D1.sam > D1_filtered.bam
- samtools sort -m 19G -o D1_sorted.bam D1_filtered.bam

Then to obtain coverages:

- bedtools genomecov -ibam D1_sorted.bam -d > D1_coverages.txt

Then to obtain SNPs and indels:

- samtools mpileup -t DP -uvf reference_genome.fa D1_sorted.bam > D1.vcf
- bcftools call -f GQ -o D1_snps.vcf -O v -V indels -cv D1.vcf

- bcftools call -f GQ -o D1_indels.vcf -O v -V snps -cv D1.vcf
- vcfutils.pl varFilter -in D1_snps.vcf > D1_snps.filt.vcf
- vcfutils.pl varFilter -in D1_indels.vcf > D1_indels.filt.vcf

References

Hamilton EP, Kapusta A, Huvos PE, Bidwell SL, Zafar N, Tang H, Hadjithomas M, Krishnakumar V, Badger JH, Caler EV, et al. 2016. Structure of the germline genome of Tetrahymena thermophila and relationship to the massively rearranged somatic genome. Elife 5.

Li H. 2018. Minimap2: pairwise alignment for nucleotide sequences. Bioinformatics 34:3094-3100.

Sheng Y, Duan L, Cheng T, Qiao Y, Stover NA, Gao S. 2020. The completed macronuclear genome of a model ciliate Tetrahymena thermophila and its application in genome scrambling and copy number analyses. Sci China Life Sci 63:1534-1542.

Walker BJ, Abeel T, Shea T, Priest M, Abouelliel A, Sakthikumar S, Cuomo CA, Zeng Q, Wortman J, Young SK, et al. 2014. Pilon: an integrated tool for comprehensive microbial variant detection and genome assembly improvement. PloS one 9:e112963.

Supplementary Material 3: Gene counts per MAC chromosome

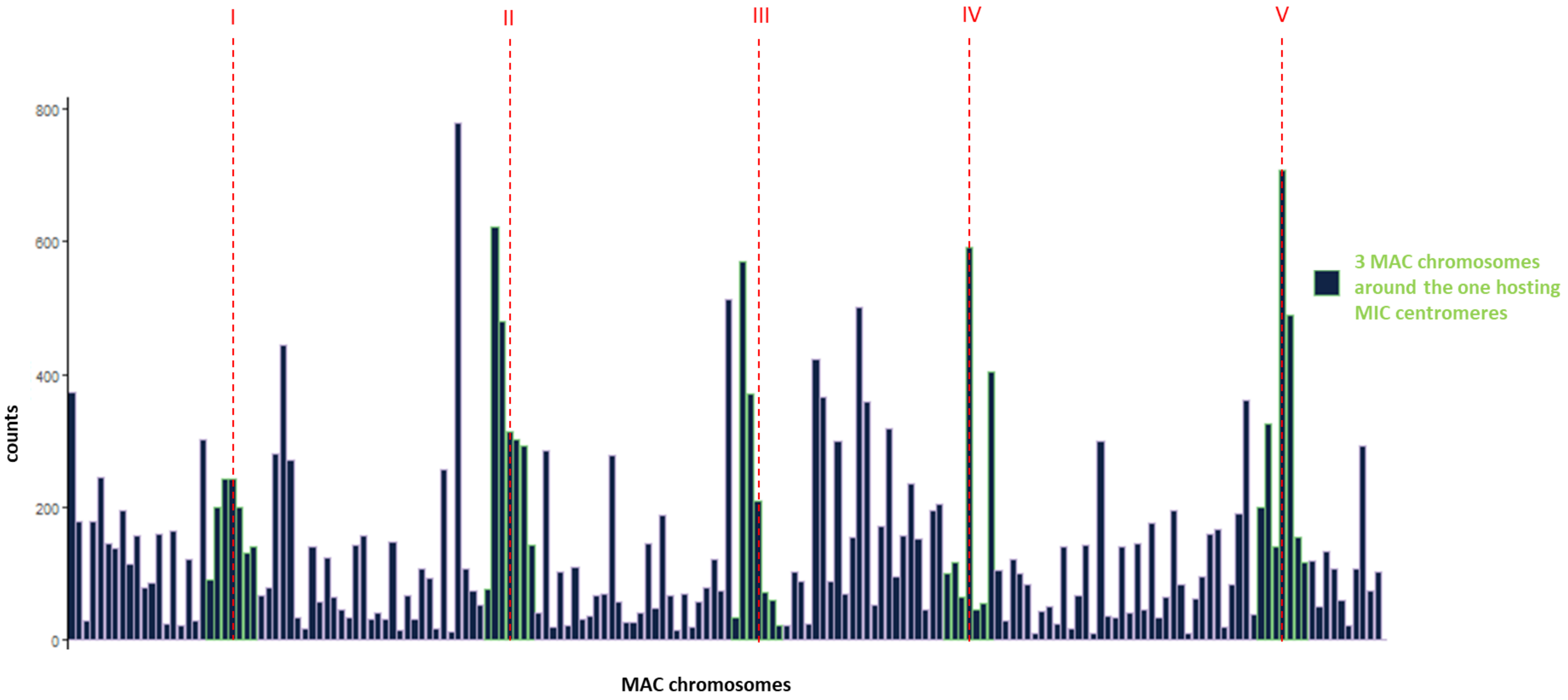

Supplementary Material 4: Neighbour-Joining tree

This analysis was based on the 22 MAC genome assemblies generated in this study (described in Supplementary Material 3) and MAC genomes assemblies downloaded from http://ciliate.ihb.ac.cn.

The genomes were analysed using andi v0.13 using default parameters (Haubold et al., 2015), and a neighbour-joining tree was built from the resulting kmer-based distances using NINJA v1.2.2 (Wheeler, 2009).

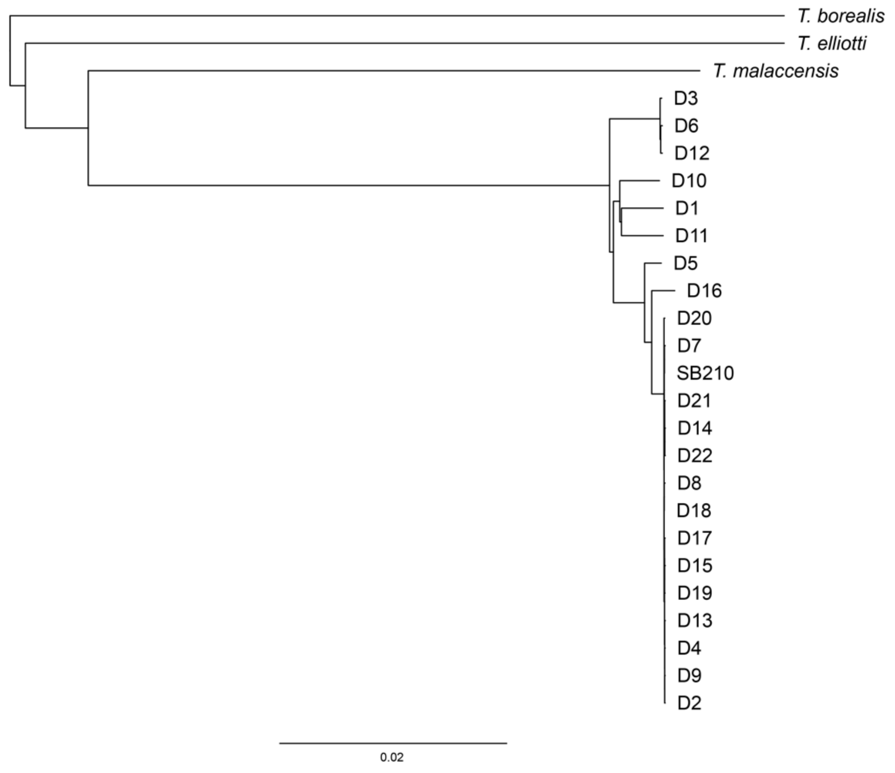

Haubold, B., Klotzl, F., and Pfaffelhuber, P. (2015). andi: fast and accurate estimation of evolutionary distances between closely related genomes. Bioinformatics *31*, 1169-1175.
Wheeler, T.J. (2009). Large-scale neighbor-joining with NINJA. In S.L. Salzberg and T. Warnow (Eds.), Proceedings of the 9th Workshop on Algorithms in Bioinformatics. WABI 2009, 375-389.

Supplementary Material 5: Number of SNPs per 100,000 nucleotides per MAC chromosome (overall strains)

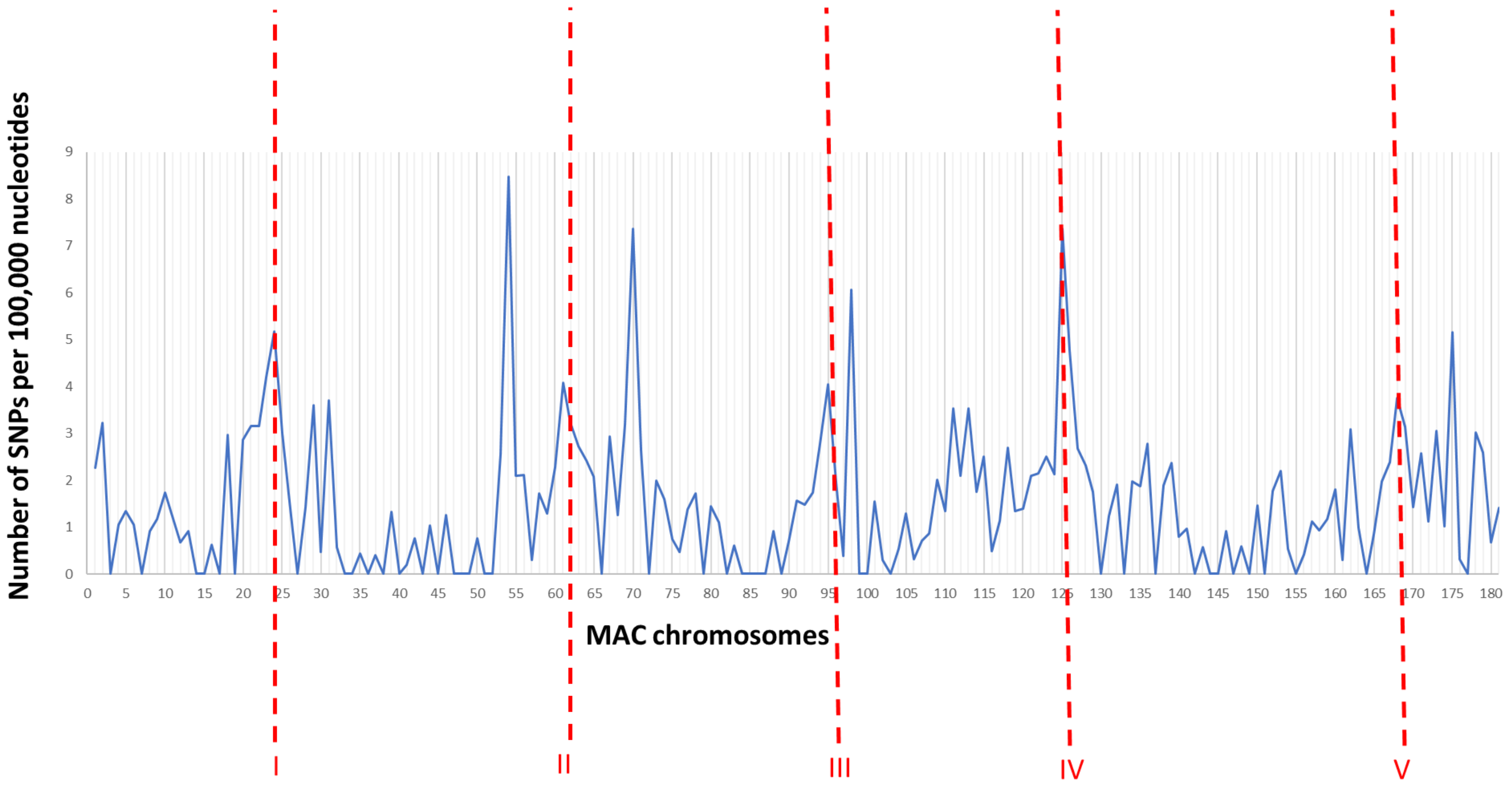

The positions of MIC centromeres are represented by the five red dashed lines.

Suplementary material 6: mitochondrial genomes

A)

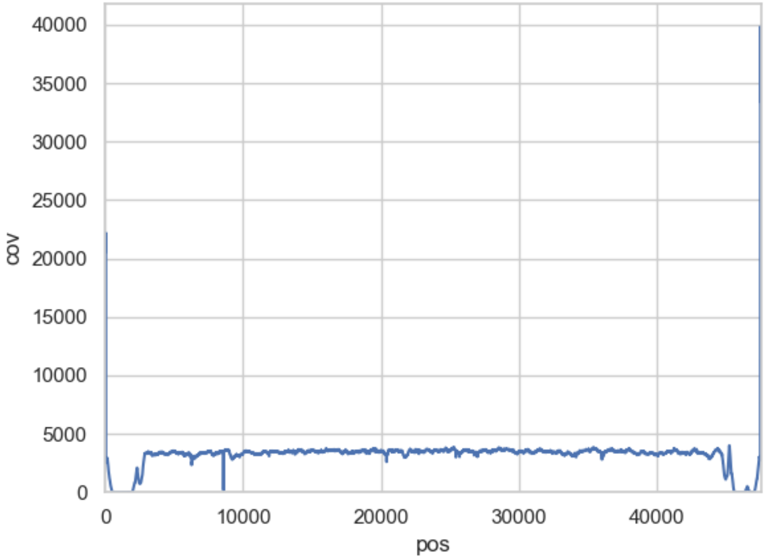

Average coverage along the mitochondrial genome over the 22 *T. thermophila* strains. The 2kb from each extremity, composed of repeat regions, were excluded.

B)

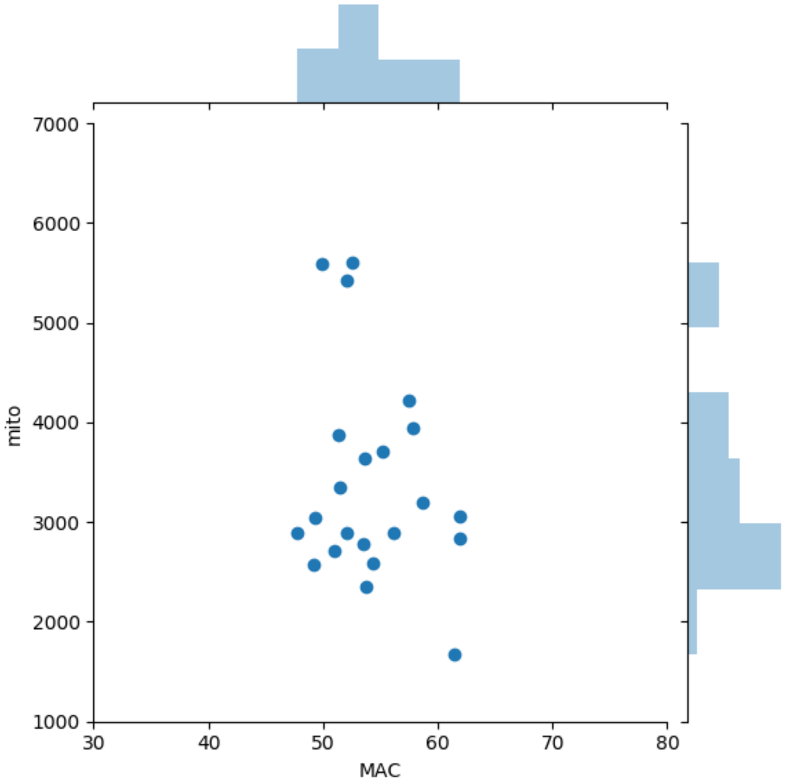

Mitochondrial coverage as a function of MAC coverage in the 22 strains, with respective distributions on the top and right of the graph.

C)

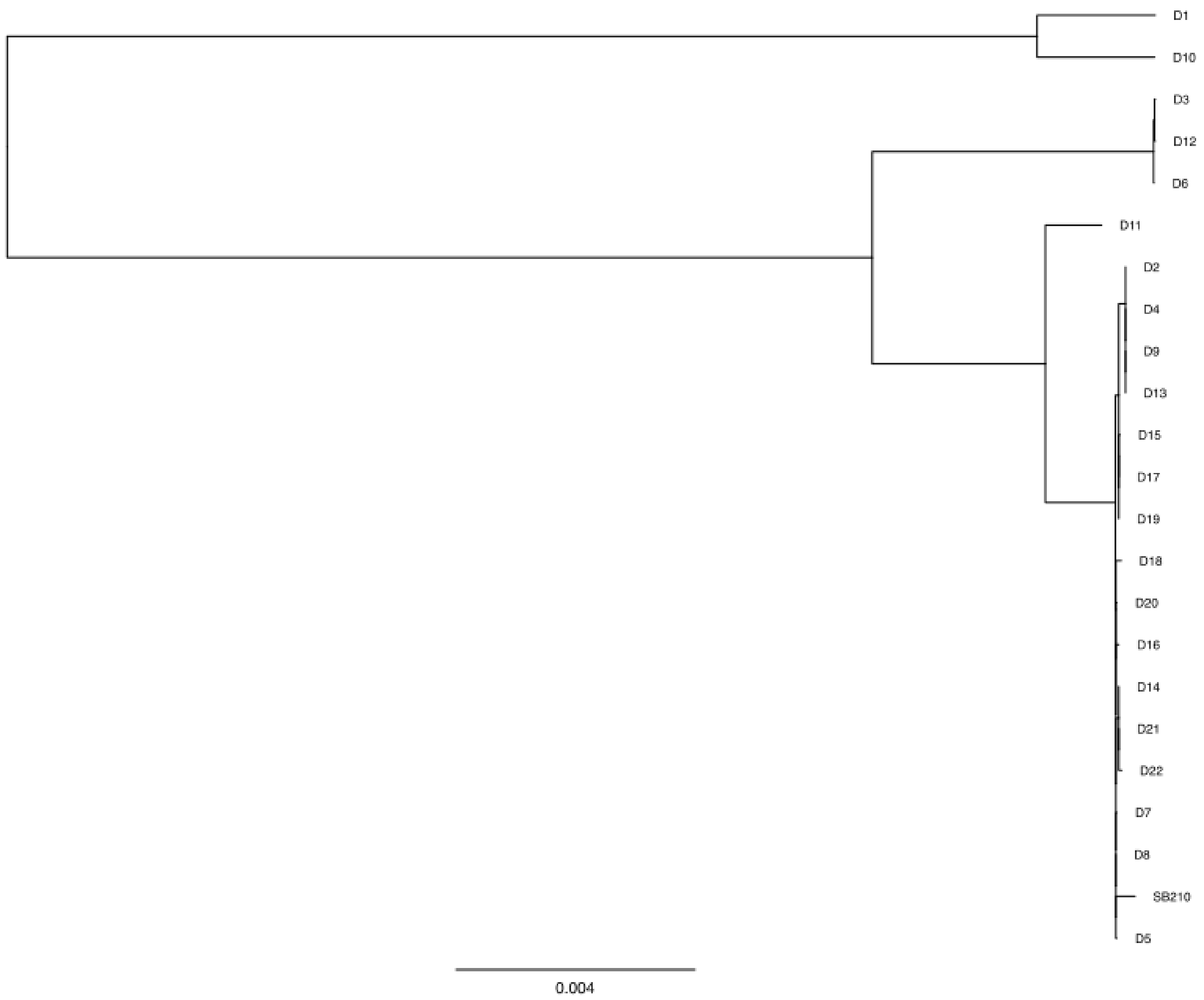

Neighbour-joining tree based on the whole mitochondrial genome of our 22 strains plus the reference SB210 mitochondrial genome.

D)

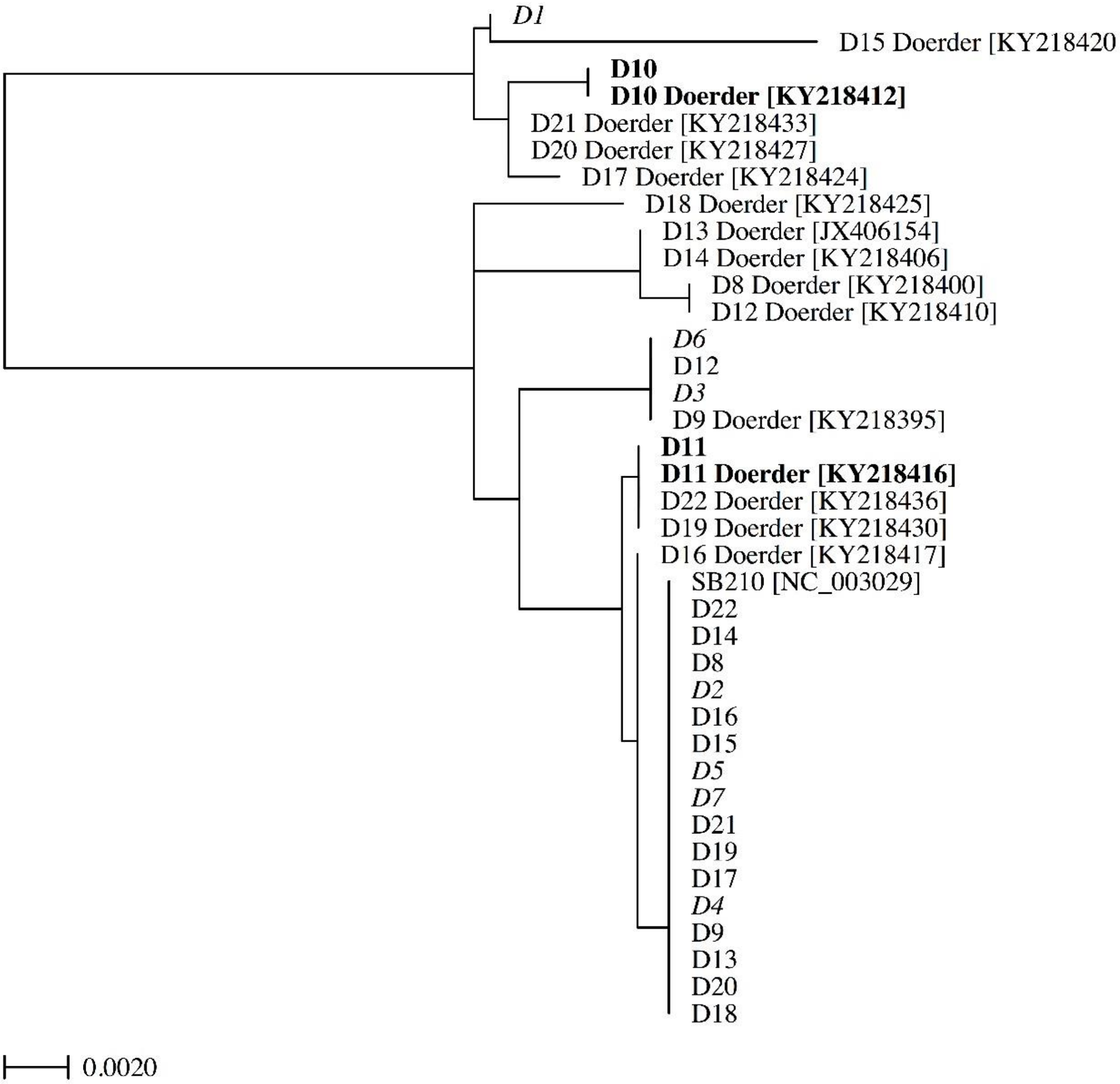

Neighbour-joining tree based on based on the Cox 1 region uniquely of our 22 strains plus the reference SB210 mitochondrial genome and the 15 common sequences between our work and those of Zufall et al. (2014) and Doerder (2019). Strains in bold indicate congruence between the two studies, strains in italic were not sequenced before our study.

References

Doerder FP. Barcodes Reveal 48 New Species of *Tetrahymena*, *Dexiostoma*, and *Glaucoma*: Phylogeny, Ecology, and Biogeography of New and Established Species. J Eukaryot Microbiol 2019;66:182–208.

Zufall RA, Dimond KL, Doerder FP. Restricted distribution and limited gene flow in the model ciliate *Tetrahymena thermophila*. Mol Ecol 2013;22:1081–1091.

Supplementary Material 7: polymorphism around MDS junction

in-line MDS

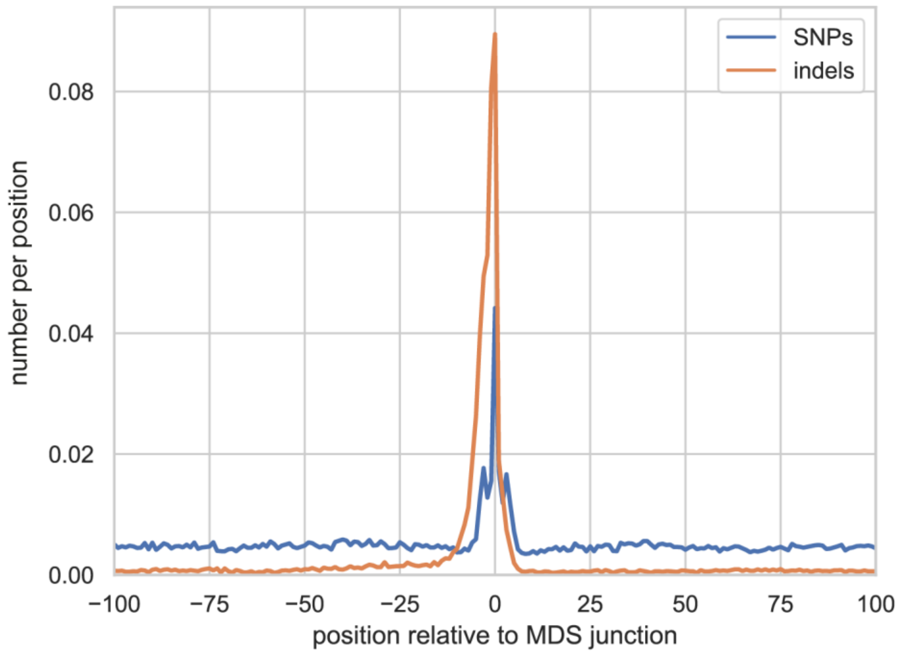

scrambled MDS

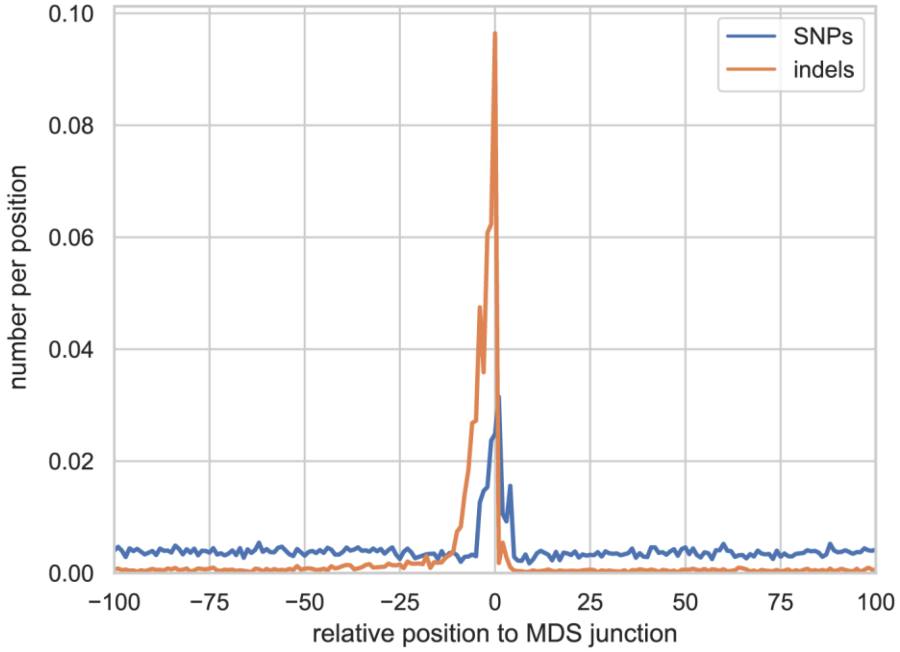

Number of SNPs and indels per position with respect to the MDS junction positions for in-line and scrambled MDS across the 22 strains

Supplementary Material 8: D18

This document attempts to demonstrate, using genomic evidences, that the strain D18 is amicronucleated (*i.e.*, absence of MIC genome). The strategy here consisted on blasting the IES (*i.e.*, sequences usually specific to the MIC genome) on the genome assembly of the different strains, which should contain both the MIC and MAC genomes, to identify the presence or absence of MIC genome.

Firstly, the trimmed reads of each of the 22 strains were assembled into contigs with SPAdes v3.14.1 (Prjibelski et al., 2020) using the options '-k 105 --only-assembler –careful'. The assembly statistics are provided in the following table:

strain	size (Mb)	N50 (kb)	nb seq	longest seq (kb)	% GC	% N
D1	108.7	325.82	1276	1534.3	22.3	0.005
D2	109.2	275.51	3231	1545.53	22.3	0.0077
D3	113	356.79	1380	2096.37	22.32	0.0042
D4	108.8	270.83	3249	1294.23	22.3	0.0057
D5	114.4	343.67	1480	1635.37	22.32	0.0056
D6	109.7	370.36	1237	1935.81	22.31	0.0047
D7	115.4	317.15	1541	1336.47	22.32	0.0056
D8	113.2	270.11	3718	1295.31	22.31	0.0053
D9	108.8	280.64	3207	1294.2	22.3	0.0067
D10	111.4	279.25	1423	1922.01	22.31	0.0047
D11	111.7	271.28	1360	1639.3	22.31	0.0051
D12	110.2	334.82	1304	1638.83	22.31	0.0054
D13	107.8	316.38	1742	1294.21	22.31	0.0064
D14	108	301.85	2238	1415.04	22.3	0.0066
D15	110.6	269.71	3670	1056.55	22.32	0.005
D16	108	328.09	2204	1645.06	22.3	0.0055
D17	110.1	286.75	3255	1294.17	22.33	0.0071
D18	103.7	345.13	998	1589.16	22.29	0.0049
D19	108.6	319.58	2045	1296.67	22.3	0.0057
D20	107	313.06	1268	1293.21	22.29	0.0053
D21	107	332.93	1497	1426.63	22.3	0.0064
D22	107.3	305.31	2044	1501.08	22.29	0.0072

We then extracted the 7,544 IES (28,529,842 positions) from the SB210 MIC genome (Hamilton et al., 2016) using an in-house script, and blasted this set of sequences on each of the 22 genome assemblies (blastn v2.6.0; options '-perc_identity 98 -evalue 1e-100').

Finally, we reported in the following table, for each strain, the number of IES having a blast hit against the genome assembly and the number of distinct IES positions matching the genome assembly:

strain	nb IES	nb IES positions
D1	1828	1123173
D2	1350	2666430
D3	2645	1770039
D4	1299	2535631
D5	4152	4062190
D6	1828	1268319
D7	4894	5306020
D8	2157	4299870
D9	1330	2579992
D10	2614	1646480
D11	2751	1809949
D12	2286	1430468
D13	1299	2068254
D14	1380	2327504
D15	1522	3019333
D16	1042	1251402
D17	1503	2900962
**D18**	**122**	**35134**
D19	1512	2554576
D20	1972	1356975
D21	1378	1963792
D22	1348	2124265

In all strains, the proportion of IES and IES positions with a blast hit were relatively low because the IES are filled with repeat sequences and transposable elements (Hamilton et al., 2016). Nevertheless, despite its high similarity to the SB210 strain (only 48 SNPs in the MAC genome), the genome assembly of the strain D18 showed a number of IES positions lower than those observed in other strains by several orders of magnitude.

We believed that the above results indicated an absence of MIC genome in the assembly of the strain D18, with the few IES detected being either retained or differentially excised in the MAC genome of this strain.

References

Hamilton, E.P., Kapusta, A., Huvos, P.E., Bidwell, S.L., Zafar, N., Tang, H., Hadjithomas, M., Krishnakumar, V., Badger, J.H., Caler, E.V., et al. (2016). Structure of the germline genome of Tetrahymena thermophila and relationship to the massively rearranged somatic genome. Elife *5*.

Prjibelski, A., Antipov, D., Meleshko, D., Lapidus, A., and Korobeynikov, A. (2020). Using SPAdes De Novo Assembler. Curr Protoc Bioinformatics *70*, e102.

Supplementary Material 9: heatmap of indels at MDS junctions as compared with SB210

This heatmap is the same as in Figure 3B, including the three datasets published in (Wang et al., 2021):

_ SRR11922748: population B

_ SRR11922749: population A

_ SRR11922750: ancestral population

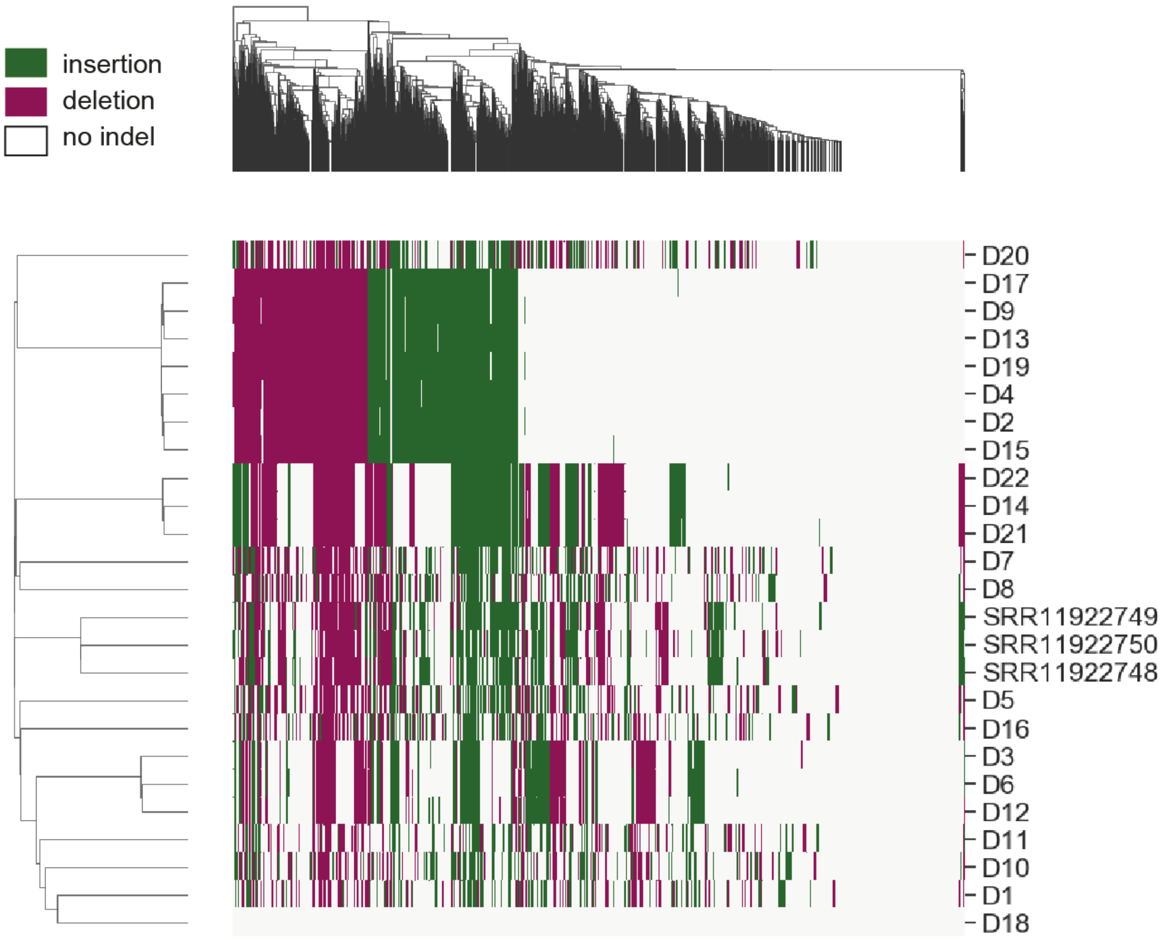

Wang, G., Fu, L., Xiong, J., Mochizuki, K., Fu, Y., and Miao, W. (2021). Identification and Characterization of Base-Substitution Mutations in the Macronuclear Genome of the Ciliate Tetrahymena thermophila. Genome Biol Evol 13.

Supplementary Material 10:
Single nucleotide diversity within strains

A)

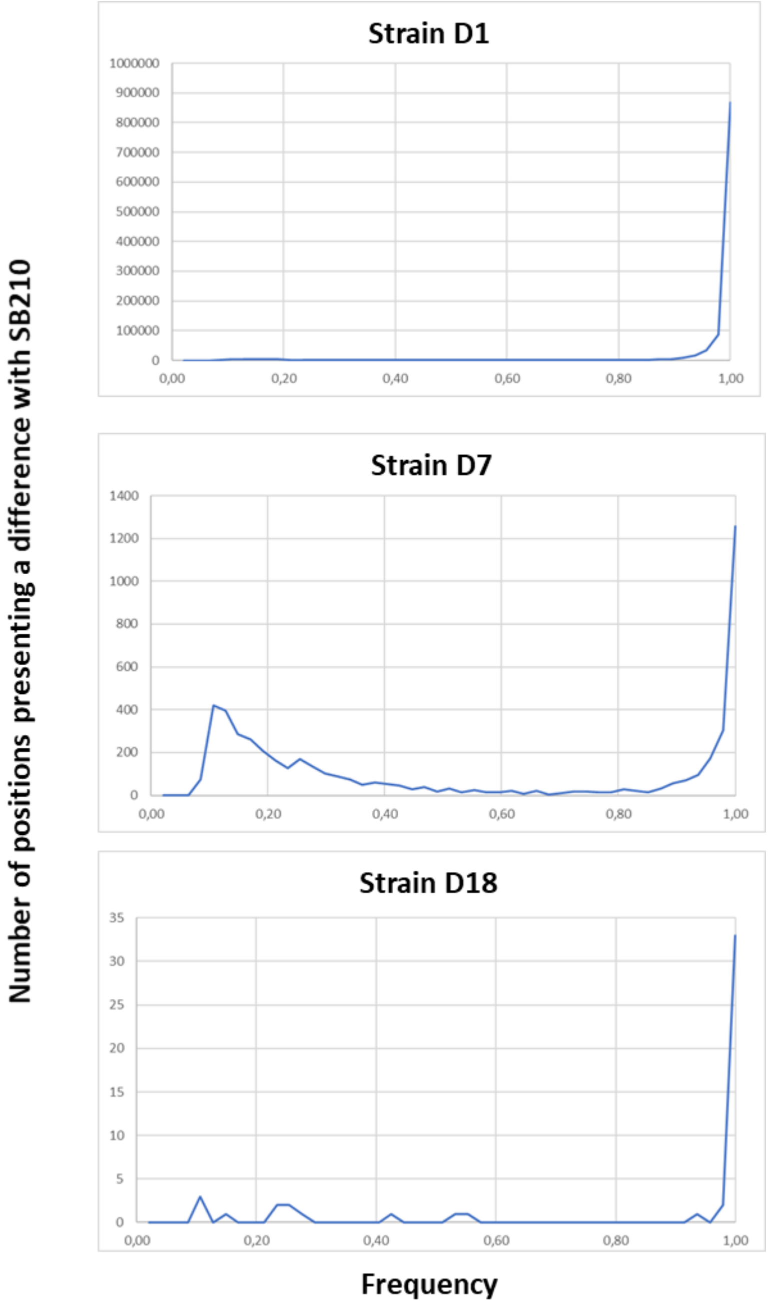

Number of positions presenting a difference with the reference genome SB210 as a function of their frequency in three strains over the 22 with decreasing divergence from SB210 genome. D1 is highly distant, D7 is closely related, and D18 is almost identical to SB210.

B)

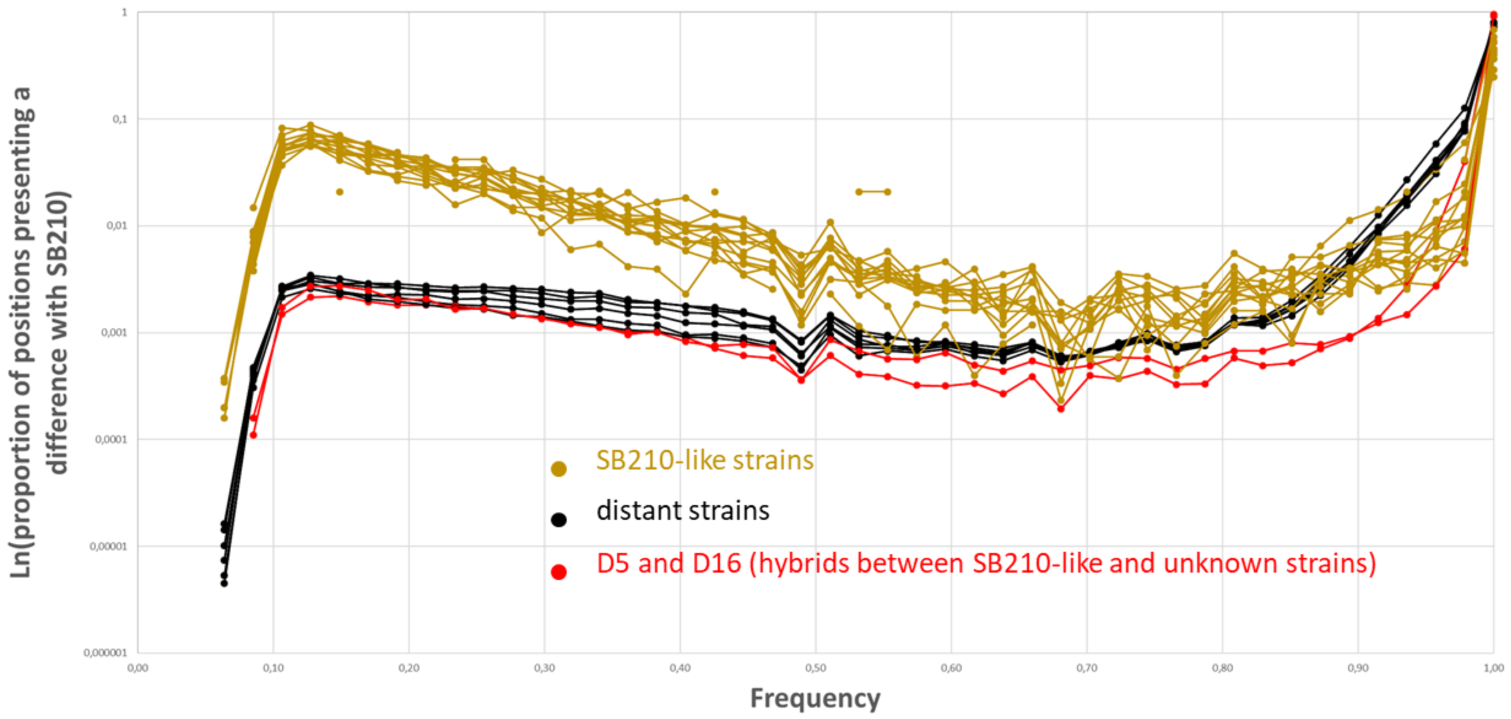

Proportion of positions presenting a difference with SB210 (calculated as the number of variable positions of a given frequency for the alternative variant over the total number of variable positions) as a function of their frequency within each strain identified for the three groups of strains (SB210-like, distant and hybrid strains).

C)

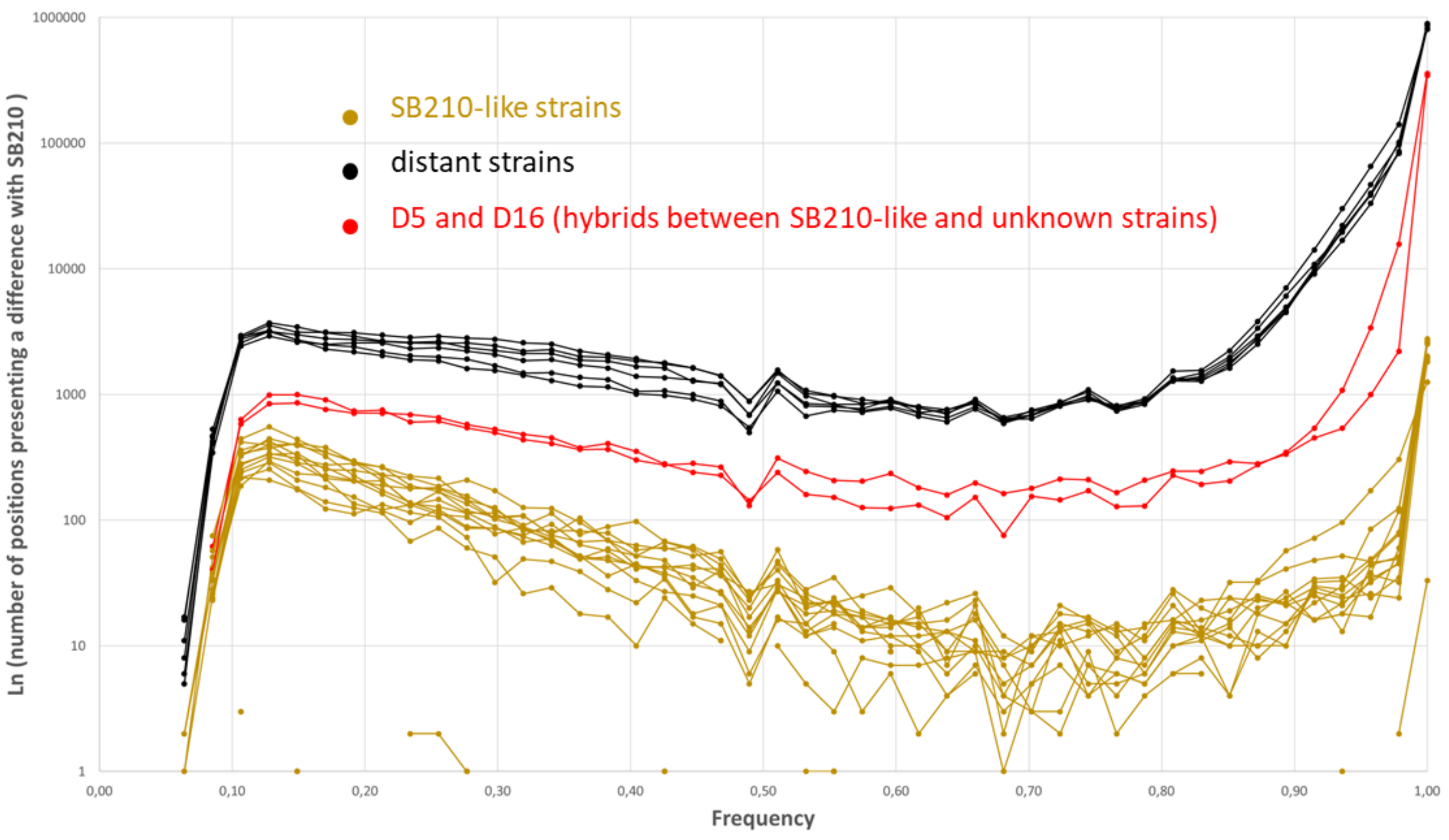

Number of positions presenting a difference with SB210 as a function of their frequency within each strain identified for three groups of strains (SB210-like, distant and hybrid strains).

Supplementary Material 11: pN/pS per GO term

Distributions of pN/pS values for each slim Gene Ontology (GO). We only kept in the following boxplots GO with at least 10 genes (the number of genes assigned to each GO is indicated within brackets). Red arrows indicate GOs discussed in the main manuscript.

Cellular component

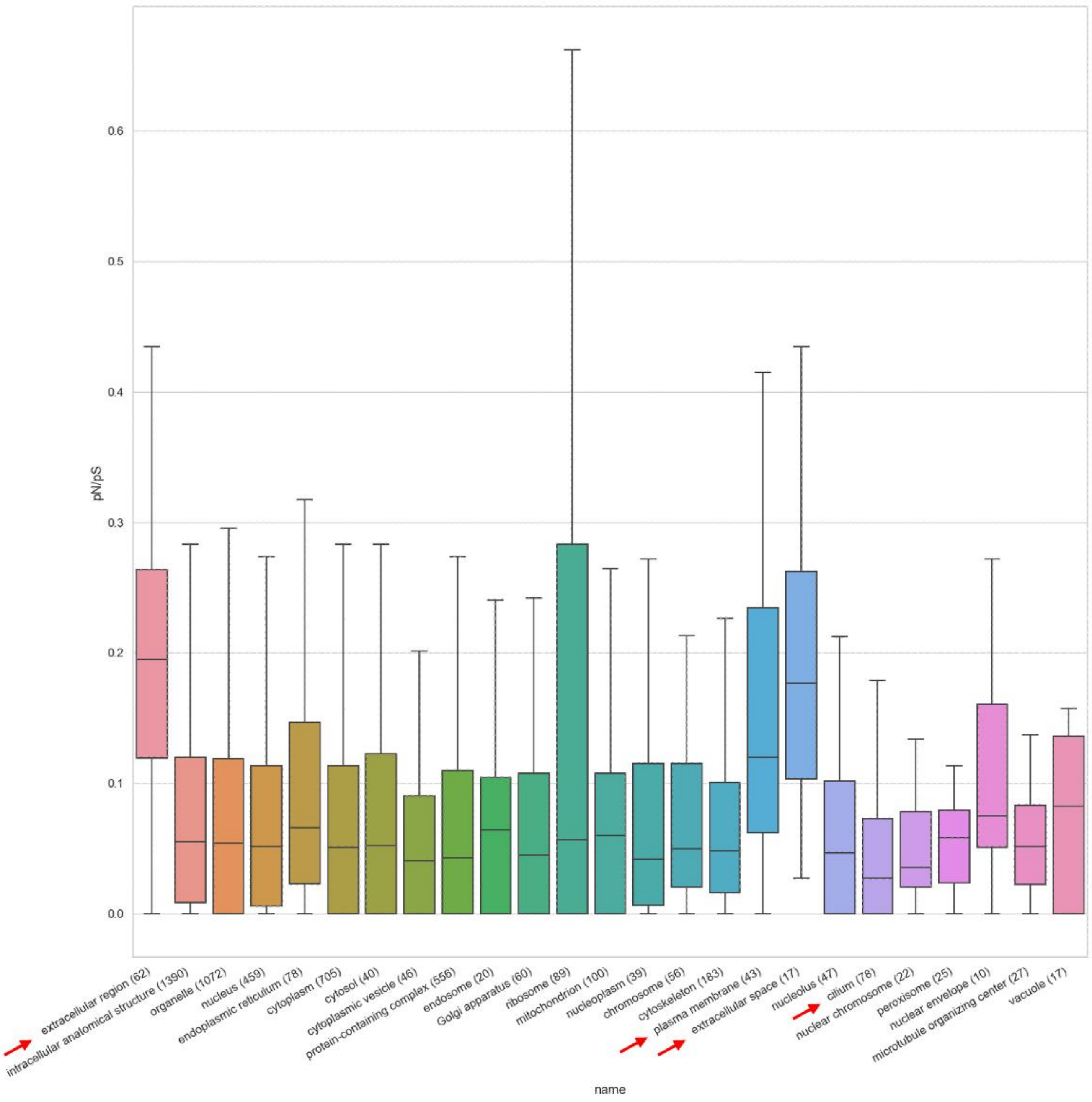

Molecular function

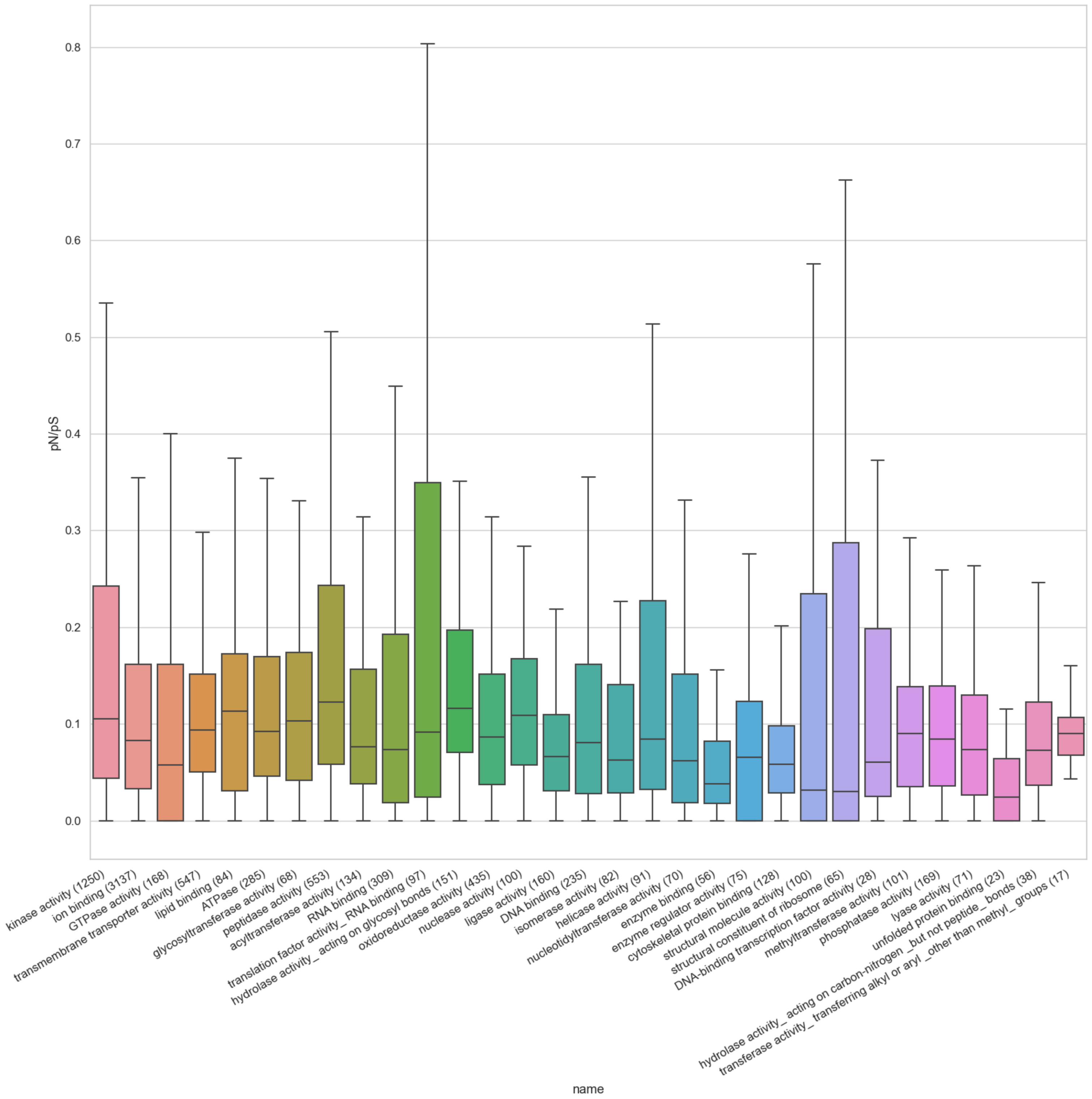

Biological process

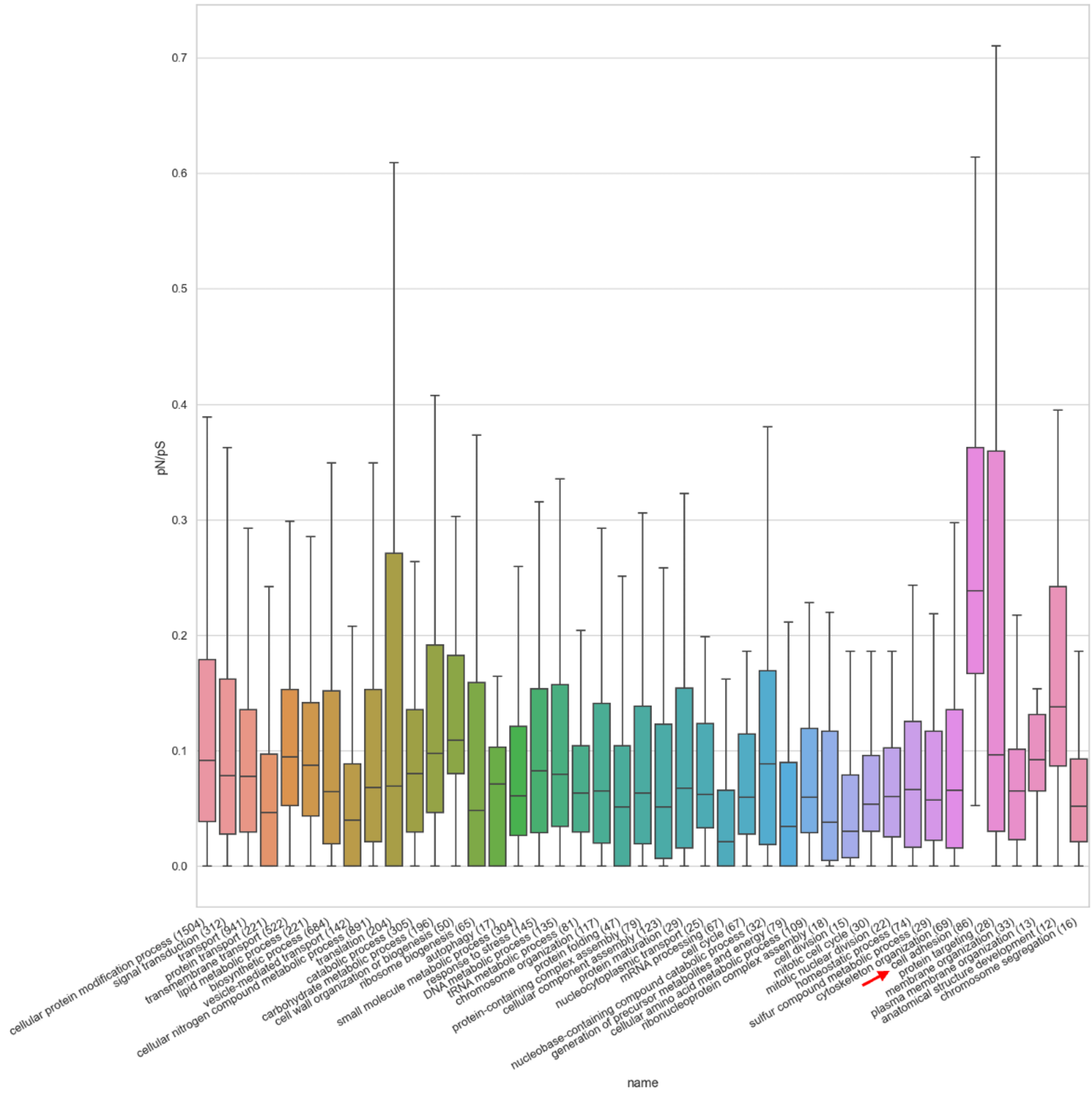

Supplementary Material 12: Direction of Selection per GO term

Distributions of DoS values for each slim Gene Ontology (GO). We only kept in the following boxplots GO with at least 10 genes (the number of genes assigned to each GO is indicated within brackets). Red arrows indicate GOs discussed in the main manuscript.

Cellular component

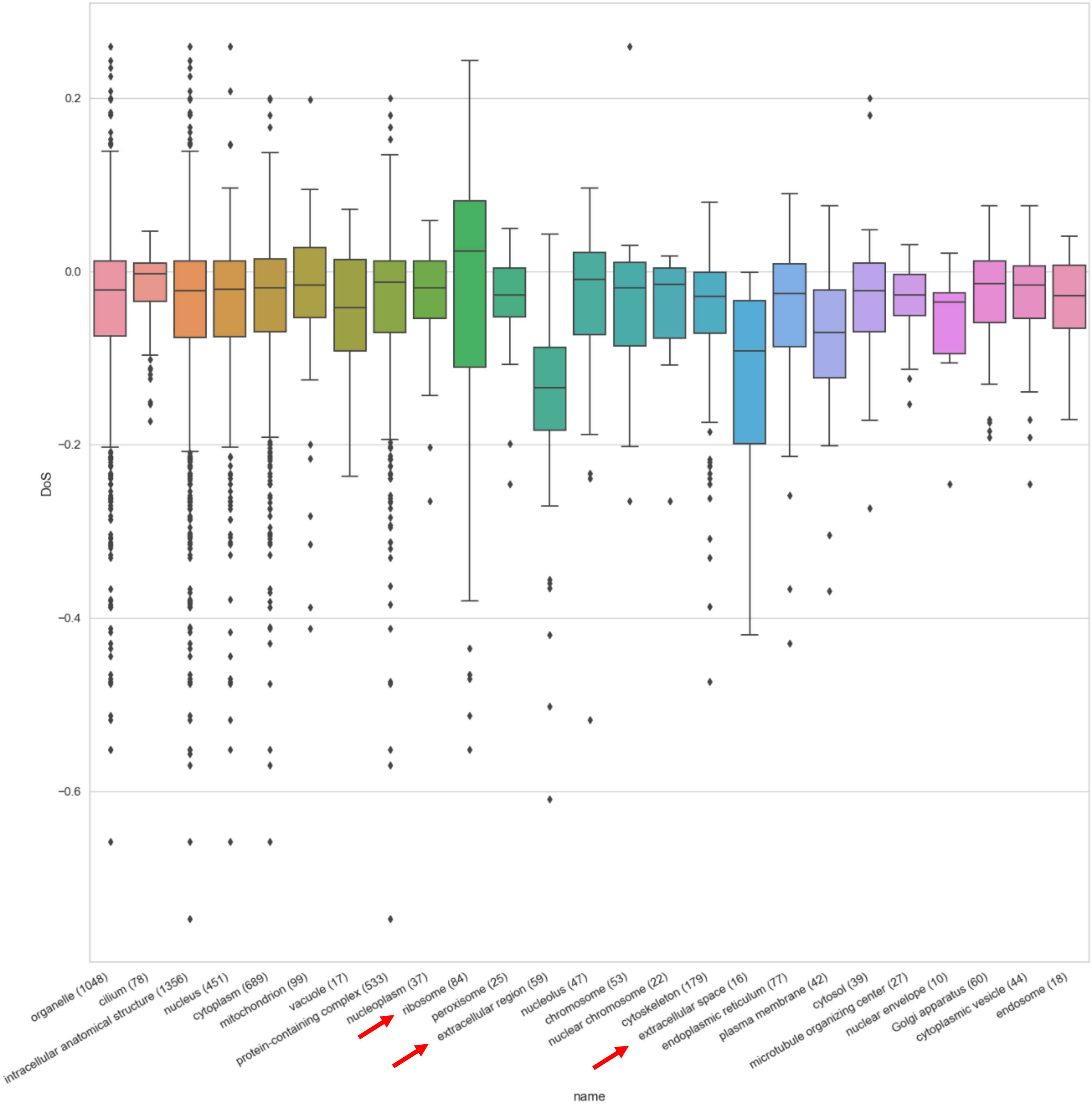

Molecular function

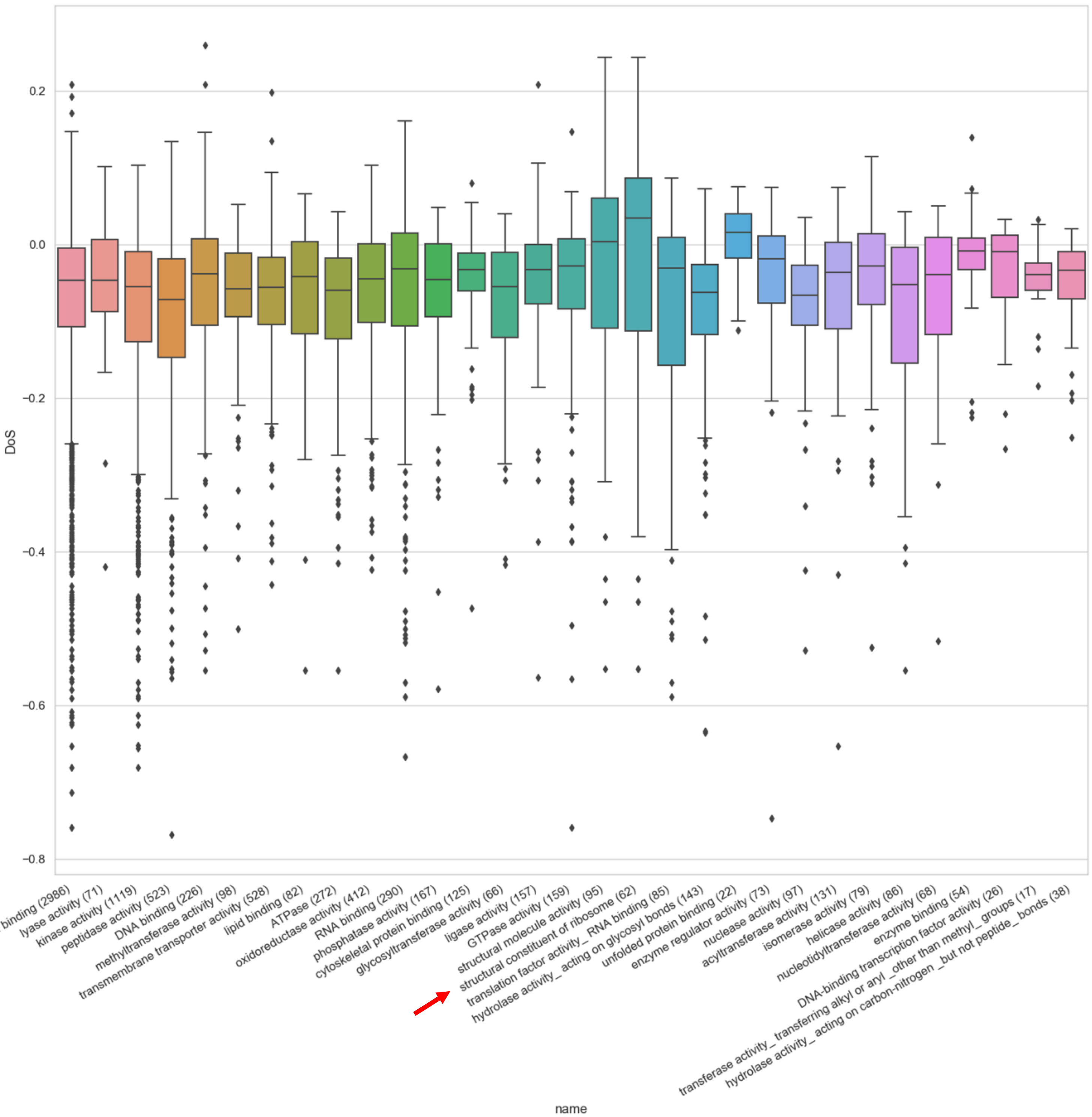

Biological process

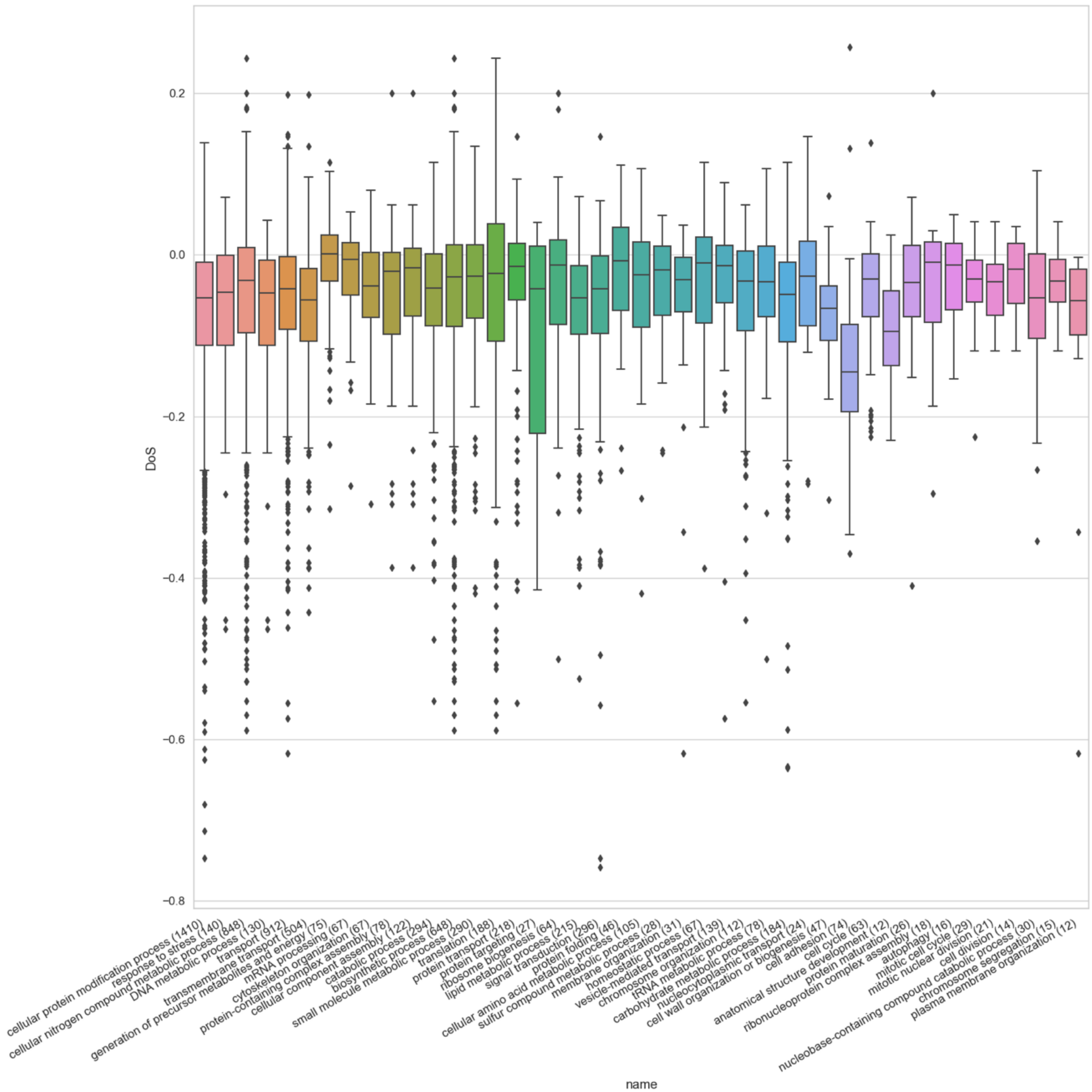

Supplementary Material 13: Direction of Selection around MIC centromeres

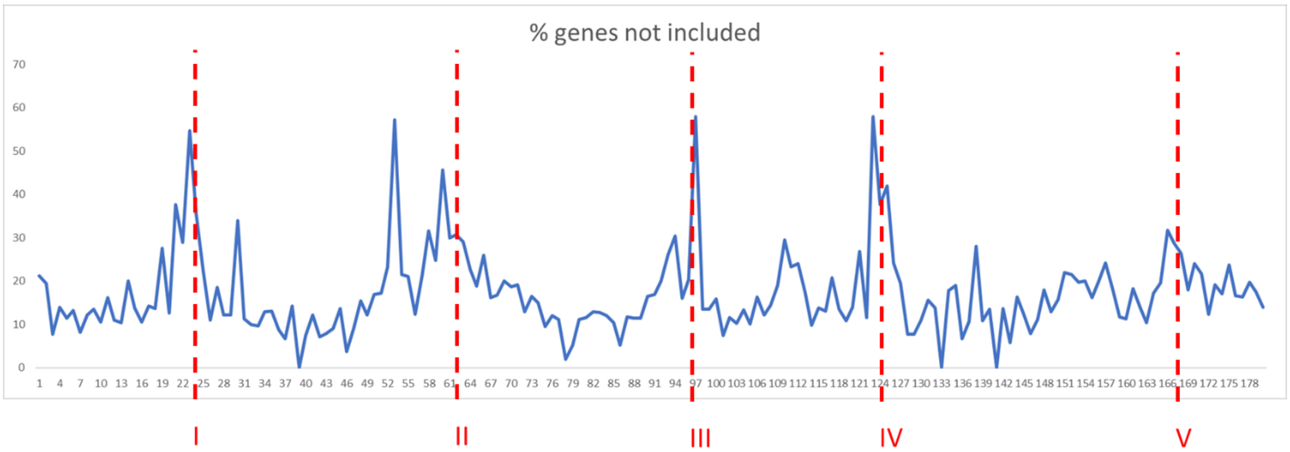

Number of genes not included in the DoS estimation because no orthologs were found in the genus *Tetrahymena*. Red dashed bars correspond to the positions of MIC centromeres.

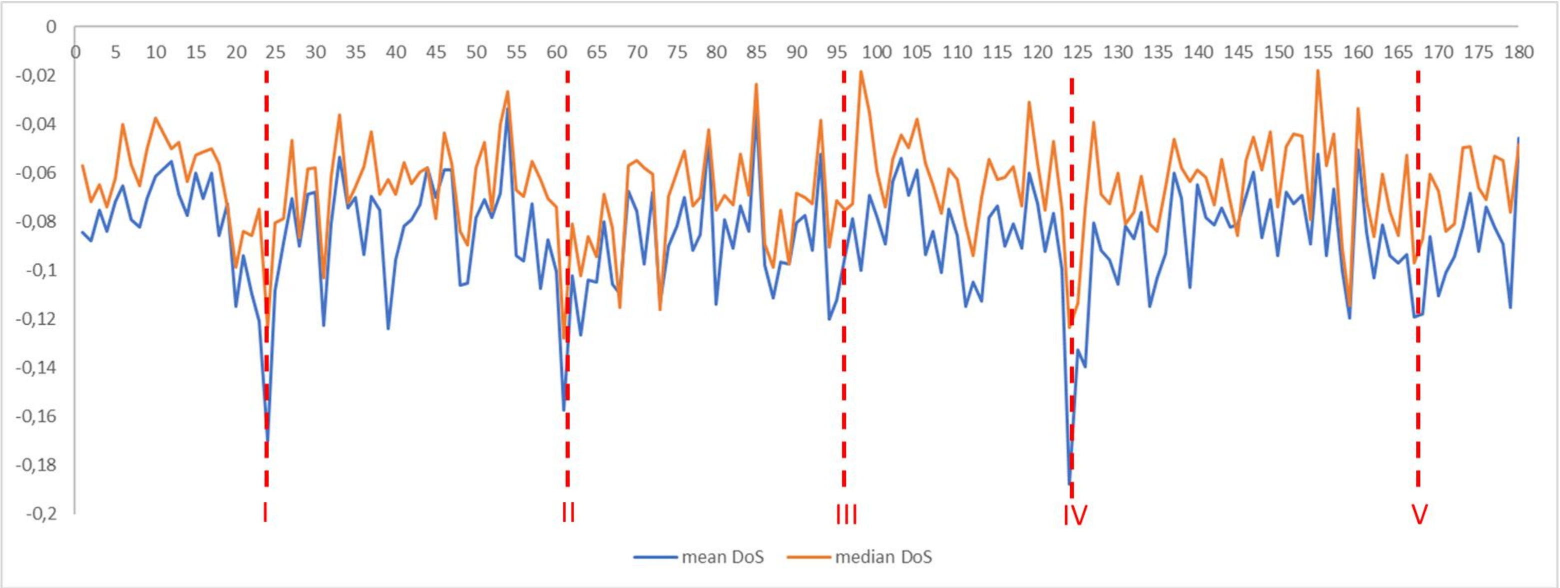

Mean (in blue) and median (in orange) of genes DoS per MAC chromosome. Red dashed bars correspond to the positions of MIC centromeres.
